# TMPRSS2 and TMPRSS4 promote SARS-CoV-2 infection of human small intestinal enterocytes

**DOI:** 10.1126/sciimmunol.abc3582

**Published:** 2020-05-13

**Authors:** Ruochen Zang, Maria Florencia Gomez Castro, Broc T. McCune, Qiru Zeng, Paul W. Rothlauf, Naomi M. Sonnek, Zhuoming Liu, Kevin F. Brulois, Xin Wang, Harry B. Greenberg, Michael S. Diamond, Matthew A. Ciorba, Sean P. J. Whelan, Siyuan Ding

**Affiliations:** ^1^Department of Molecular Microbiology, Washington University School of Medicine, St. Louis, MO, USA. ^2^Key Laboratory of Marine Drugs, Ministry of Education, Ocean University of China, Qingdao, China. ^3^Department of Medicine, Division of Infectious Diseases, Washington University School of Medicine, St. Louis, MO, USA. ^4^Program in Virology, Harvard Medical School, 200 Longwood Ave, Boston, MA, USA. ^5^Department of Medicine, Division of Gastroenterology, Washington School of Medicine, St. Louis, MO, USA. ^6^Department of Pathology, Stanford School of Medicine, Stanford, CA, USA. ^7^VA Palo Alto Health Care System, Department of Veterans Affairs, Palo Alto, CA, USA. ^8^Department of Medicine, Division of Gastroenterology and Hepatology, and Department of Microbiology and Immunology, Stanford School of Medicine, Stanford, CA, USA. ^9^Department of Pathology and Immunology, Washington University School of Medicine, St. Louis, MO, USA.

## Abstract

Gastrointestinal symptoms and fecal shedding of SARS-CoV-2 RNA are frequently observed in COVID-19 patients. However, it is unclear whether SARS-CoV-2 replicates in the human intestine and contributes to possible fecal-oral transmission. Here, we report productive infection of SARS-CoV-2 in ACE2^+^ mature enterocytes in human small intestinal enteroids. Expression of two mucosa-specific serine proteases, TMPRSS2 and TMPRSS4, facilitated SARS-CoV-2 spike fusogenic activity and promoted virus entry into host cells. We also demonstrate that viruses released into the intestinal lumen were inactivated by simulated human colonic fluid, and infectious virus was not recovered from the stool specimens of COVID-19 patients. Our results highlight the intestine as a potential site of SARS-CoV-2 replication, which may contribute to local and systemic illness and overall disease progression.

## Teaser

The SARS-CoV-2 virus infects cultured ACE2-expressing human enterocytes aided by the TMPRSS2 and TMPRSS4 serine proteases.

## Introduction

Coronavirus disease 2019 (COVID-19) has emerged as a new world pandemic, infecting millions and causing substantial morbidity and mortality. This outbreak is caused by a novel severe acute respiratory syndrome coronavirus, SARS-CoV-2 (*1*, *2*), which belongs to the family of *Coronaviridae*, a group of enveloped, non-segmented, positive-sense RNA viruses. Currently, there are no clinically approved countermeasures available for COVID-19. Severe SARS-CoV-2-associated disease occurs more frequently in the elderly and those with specific comorbidities (e.g., diabetes and obesity), although the basis for this remains largely unknown. SARS-CoV-2 also has a more protracted disease time course and prolonged viral RNA shedding compared to some other respiratory viruses, raising questions about other potential modes of transmission.

The attachment of SARS-CoV-2 to the target cell is initiated by interactions between the spike glycoprotein (S) and its cognate receptor, angiotensin converting enzyme 2 (ACE2) (*2*–*5*). Following receptor engagement, SARS-CoV-2 S is processed by a plasma membrane-associated type II transmembrane serine protease, TMPRSS2, prior to membrane fusion which is essential to release the viral contents into the host cell cytosol (*3*, *6*). Both ACE2 and TMPRSS2 are expressed highly in the gastrointestinal (GI) tract, in particular by intestinal epithelial cells (IECs), the predominant target cells for many human enteric viruses. Indeed, several animal CoVs are natural enteric pathogens, cause GI diseases, and spread by the fecal-oral route (*7*). Notable GI symptoms including abdominal pain and diarrhea have been observed in 20 to 50% of COVID-19 patients, and sometimes precede the development of respiratory illness (*8*–*10*). In a US cohort-based study, 61% of patients exhibited GI symptoms (*11*). Substantial amounts of SARS-CoV-2 RNA are detected in stool specimens from COVID-19 patients (*12*–*16*). Infectious virus was not isolated from the feces of COVID-19 patients in a recent systematic study (*17*) although two studies report virus isolation (*18*, *19*). The fecal shedding data raises the possibility that SARS-CoV-2 could potentially be transmitted via the fecal-oral route (*20*).

In the present study, we aimed to address the following questions: (1) does SARS-CoV-2 infect human IECs? 2) if so, what host factors mediate efficient replication? 3) are there infectious viruses shed in the fecal samples of COVID-19 patients? Here, we present evidence that SARS-CoV-2 infects human mature enterocytes from the apical surface and triggers epithelial cell fusion. TMPRSS2 and TMPRSS4 serine proteases mediate this process by inducing cleavage of the S protein and enhancing membrane fusion. We also found that simulated human colonic fluid rapidly inactivates SARS-CoV-*2 in vitro*, rendering viral RNA noninfectious in the stool specimens. Our findings have mechanistic and translational relevance and enhance our understanding of COVID-19 pathogenesis.

## Results

### SARS-CoV-2 infects human intestinal enteroids

**Fig. 1:**
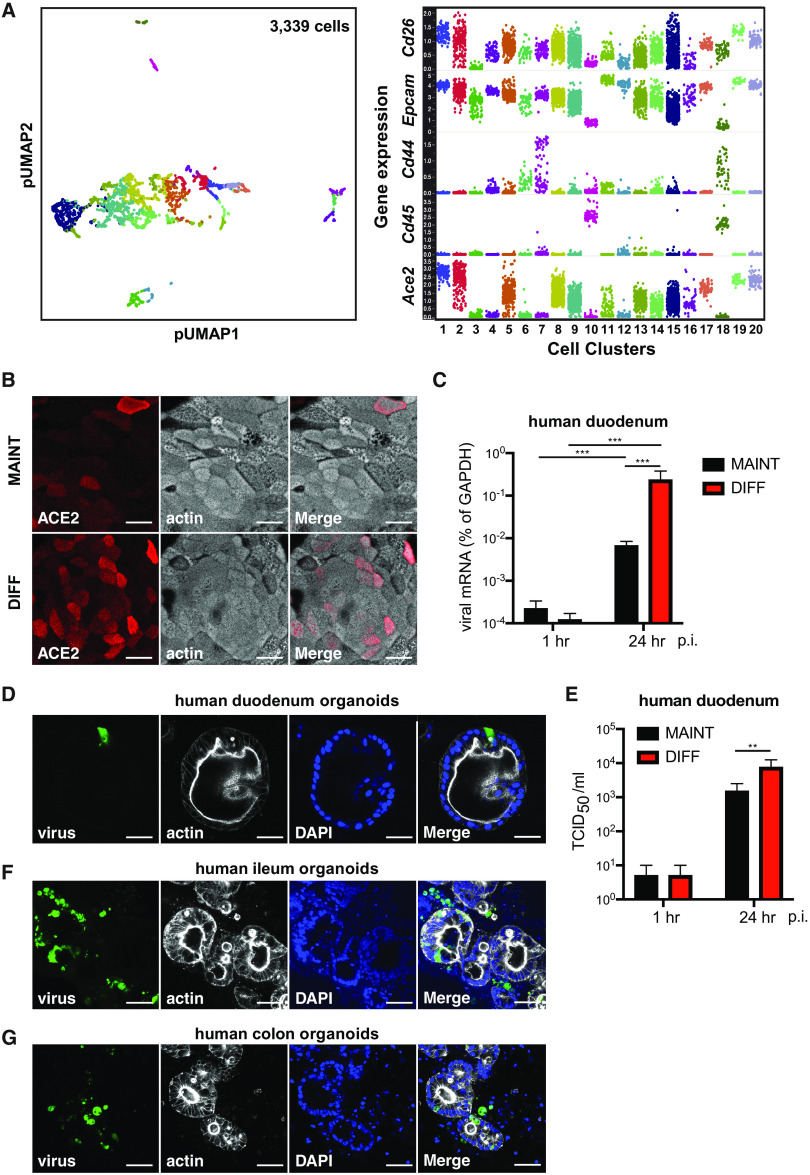
VSV-SARS-CoV-2 infects human small intestinal enteroids. Mouse small intestinal cells were analyzed by single-cell RNA-sequencing and resolved into 20 clusters based on gene expression profiles (left panel). Transcript levels of *Cd26*, *Epcam*, *Cd44*, *Cd45*, and *Ace2* were indicated for different intestinal cell subsets. Clusters 10 and 18: intraepithelial lymphocytes; clusters 1, 2, 5, 8, 9, 17, 19, 20: enterocytes; cluster 3: goblet cells; cluster 4: entero-endocrine cells; cluster 7: Tuft cells; cluster 11: crypt stem cells; cluster 12: Paneth cells. Each dot represents a single cell. Note that *Ace2*^high^ cells are also positive for *Cd26* and *Epcam* but negative for *Cd44* and *Cd45*. (A) Human duodenum enteroids were cultured in the Transwell monolayer system using maintenance (MAINT) or differentiation (DIFF) conditions for 3 days. Monolayers were stained for ACE2 (red) and actin (phalloidin, white). Scale bar: 32 μm. (B) Human duodenum enteroids in monolayer, cultured in either maintenance (MAINT) or differentiation (DIFF) conditions, were apically infected with 1.5X10^5^ plaque forming units (PFU) of VSV-SARS-CoV-2 (MOI=0.3) for 24 hours. The expression of VSV-N was measured by RT-qPCR and normalized to that of *GAPDH*. (C) Human duodenum enteroids in 3D Matrigel were cultured in maintenance (MAINT) media or differentiation (DIFF) media for 3 days and infected with 2.2X10^5^ PFU of VSV-SARS-CoV-2 for 18 hours. Enteroids were stained for virus (green), actin (phalloidin, white), and nucleus (DAPI, blue). Scale bar: 50 μm. (D) Same as (C) except that virus titers were measured using an TCID50 assay instead of viral RNA levels by QPCR. (E) Same as (D) except that human ileum enteroids were used instead. Scale bar: 80 μm. (F) Same as (D) except that human colon enteroids were used instead. Scale bar: 80 μm. For all figures except A, experiments were repeated at least three times with similar results. Figure 1A was performed once with small intestinal tissues pooled from three mice. Data are represented as mean ± SEM. Statistical significance is indicated (*p≤0.05; **p≤0.01; ***p≤0.001).

In the intestine, ACE2 functions as a chaperone for the sodium-dependent neutral amino acid transporter B^0^AT1 (encoded by *SLC6A19*) on IECs and regulates microbial homeostasis (*21*, *22*). ACE2 expression is substantially higher in the small intestine than all the other organs including the lung in both humans and mice (Fig. S1A-B). Given these data, we assessed whether SARS-CoV-2 can infect IECs as a first step for understanding its implications for fecal-oral transmission. We performed single-cell RNA-sequencing (RNA-seq) to capture the global transcriptomics in all IEC subsets in the mouse small intestinal epithelium (Fig. 1A, *left panel*). ACE2 mRNA was predominantly seen in *Cd26*^+^*Epcam*^+^*Cd44*^-^*Cd45*^-^ mature enterocytes (*23*, *24*) (Fig. 1A, *right panel*). In addition, bulk RNA-seq revealed that primary human ileum enteroids had substantially higher mRNA levels of all known CoV receptors including ACE2 than the colonic epithelial cell line HT-29 and other non-IEC human cell lines (Fig. S1C). Quantitative PCR confirmed abundant ACE2 transcript levels in both human duodenum- and ileum-derived enteroids (Fig. S1D). ACE2 protein co-localized with actin at the apical plasma membrane of human enteroid monolayers (Fig. 1B). Higher ACE2 levels correlated with the maturity of enterocytes present in differentiated enteroids (Fig. 1B), consistent with our scRNA-seq findings (Fig. 1A). In 3D Matrigel embedded enteroids, ACE2 had an almost perfect colocalization with villin (Fig. S1E), an IEC marker that localizes most strongly to the brush border of the intestinal epithelium.

Using a chimeric vesicular stomatitis virus (VSV) GFP reporter virus in which the native glycoprotein (G) is genetically replaced with SARS-CoV-2 S protein, we investigated SARS-CoV-2 S-dependent cell tropism and entry. Levels of viral mRNA increased by ~10,000 fold in human duodenum enteroids over 24 hours after infection (Fig. 1C). Differentiated enteroids with higher ACE2 levels (Fig. 1B) supported 39-fold higher VSV-SARS-CoV-2 replication than stem cell-based cultures (Fig. 1C). We visualized GFP-positive infected cells in duodenum enteroids in both traditional 3D (Fig. 1D) and in flipped “inside-out” models where the apical side of the IECs was on the outside of the spherical organoid (*25*) (Fig. S1F). In addition to viral RNA and protein, infectious virus levels were increased >1,000 fold within the intestinal epithelium at 24 hours post-infection (Fig. 1E). Robust viral replication was confirmed in human ileum- and colon-derived enteroids (Fig. 1F-G).

### SARS-CoV-2 actively replicates in ACE2+ human mature enterocytes

**Fig. 2:**
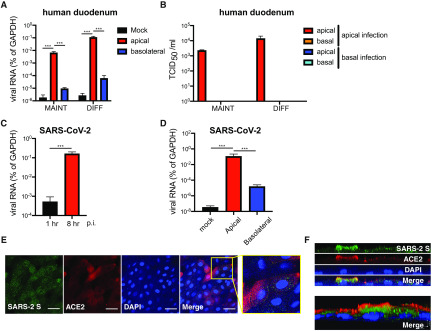
VSV-SARS-CoV-2 and wild-type SARS-CoV-2 replicate in ACE2^+^ human mature enterocytes. (A) Human duodenum enteroids in monolayer, cultured in either maintenance (MAINT) or differentiation (DIFF) conditions, were apically or basolaterally infected with 1.5X10^5^ PFU of VSV-SARS-CoV-2 for 24 hours. The expression of VSV-N was measured by RT-qPCR and normalized to that of *GAPDH*. (B) Supernatants in both apical and basal chambers were collected from (A) and were subjected to a TCID50 assay to measure the amount of infectious virus. (C) Differentiated duodenum enteroids in monolayer were apically infected with 2.5X10^5^ PFU of infectious SARS-CoV-2 virus (MOI=0.5) for 8 hours. The expression of SARS-CoV-2 N was measured by RT-qPCR using a Taqman assay and normalized to that of *GAPDH*. (D) Differentiated ileum enteroids in monolayer were apically or basolaterally infected with 2.5X10^5^ PFU of infectious SARS-CoV-2 virus (MOI=0.5) for 8 hours. The expression of SARS-CoV-2 N was measured by RT-qPCR using a Taqman assay and normalized to that of GAPDH. (E) Same as (C) except that enteroids were fixed and stained for SARS-CoV-2 S (green), ACE2 (red), and nucleus (DAPI, blue). Scale bar: 32 μm. SARS-CoV-2 infected ACE2 positive cells are enlarged in the inset (yellow box). (F) SARS-CoV-2 infected duodenum monolayers were imaged along the z-stacks and sectioned for YZ planes (top panel) and reconstructed for 3D images (bottom panel). For all figures except C, D, and E, experiments were repeated at least three times with similar results. Figure 2C, 2D, and 2E were performed twice with technical duplicates in each experiment. Data are represented as mean ± SEM. Statistical significance is indicated (*p≤0.05; **p≤0.01; ***p≤0.001).

To further characterize the potential SARS-CoV-2 route of infection, we employed the 2D monolayer system (*26*), with a clear polarity and separation of apical and basal sides that represent the luminal and lamina propria compartments, respectively. Human IECs were preferentially (> 1,000-fold) infected by VSV-SARS-CoV-2 from the apical surface compared to the basolateral side (Fig. 2A). These data are consistent with apical ACE2 expression (Fig. 1B and S1E). The newly produced virus progeny were released predominantly from the apical side into the lumen (Fig. 2B), suggesting a possibility of viral shedding and accumulation in feces in COVID-19 patients.

To define the specific IEC subset(s) targeted by SARS-CoV-2, we infected duodenal enteroids with the VSV-SARS-CoV-2 and co-stained with different IEC markers. GFP signal was found principally in CD26^+^ mature villous absorptive enterocytes (Fig. S2A). To a lesser extent, VSV-SARS-CoV-2 virus also replicated in undifferentiated enteroids (Fig. 1C, 1E, and 2A), suggesting that transit-amplifying cells or intestinal stem cells support some level of infection. Using a clinical isolate of the wild-type infectious SARS-CoV-2 virus, we confirmed that viral RNA increased by 320-fold within the first 8 hours of infection (Fig. 2C). Mirroring the VSV-SARS-CoV-2 virus, wild-type SARS-CoV-2 infection also occurred preferentially (> 10,000-fold) at the apical surface of the intestinal epithelium compared to the basolateral side (Fig. 2D). In addition, SARS-CoV-2 infection of enteroid monolayers suggested that ACE2^+^ mature enterocytes were the predominant target cells in the gut epithelium (Fig. 2E). Staining of S protein revealed interesting punctate-like structures within the infected IECs (Fig. 2E). A 3D reconstruction of confocal images showed that the S protein was concentrated toward the apical surface (Fig. 2F), suggesting a potential mechanism of polarized viral assembly and subsequent apical release. Compared to rotavirus, a prototypic enteric pathogen that is known to trigger interferon (IFN) signaling (*23*), wild-type SARS-CoV-2 infection induced a comparably robust type I IFN (IFN-β) and type III IFN (IFN-λ) expression in human IECs, from both apical and basolateral infections (Fig. S2B).

Similar to wild-type SARS-CoV-2 that induces syncytia formation (*27*), VSV-SARS-CoV-2 infection was associated with cell-cell fusion (Fig. S2C), causing syncytia formation between primary IECs in both 2D monolayer (Fig. S2D) and in 3D Matrigel (Fig. S2E and Video S1). The cell fusion may lead to subsequent cytopathic effect and a breach of the intestinal epithelium integrity, and thus may have implications regarding the common GI symptoms seen in COVID-19 patients (*28*–*32*).

### TMPRSS2, TMPRSS4 but not ST14 promote SARS-CoV-2 entry

**Fig. 3:**
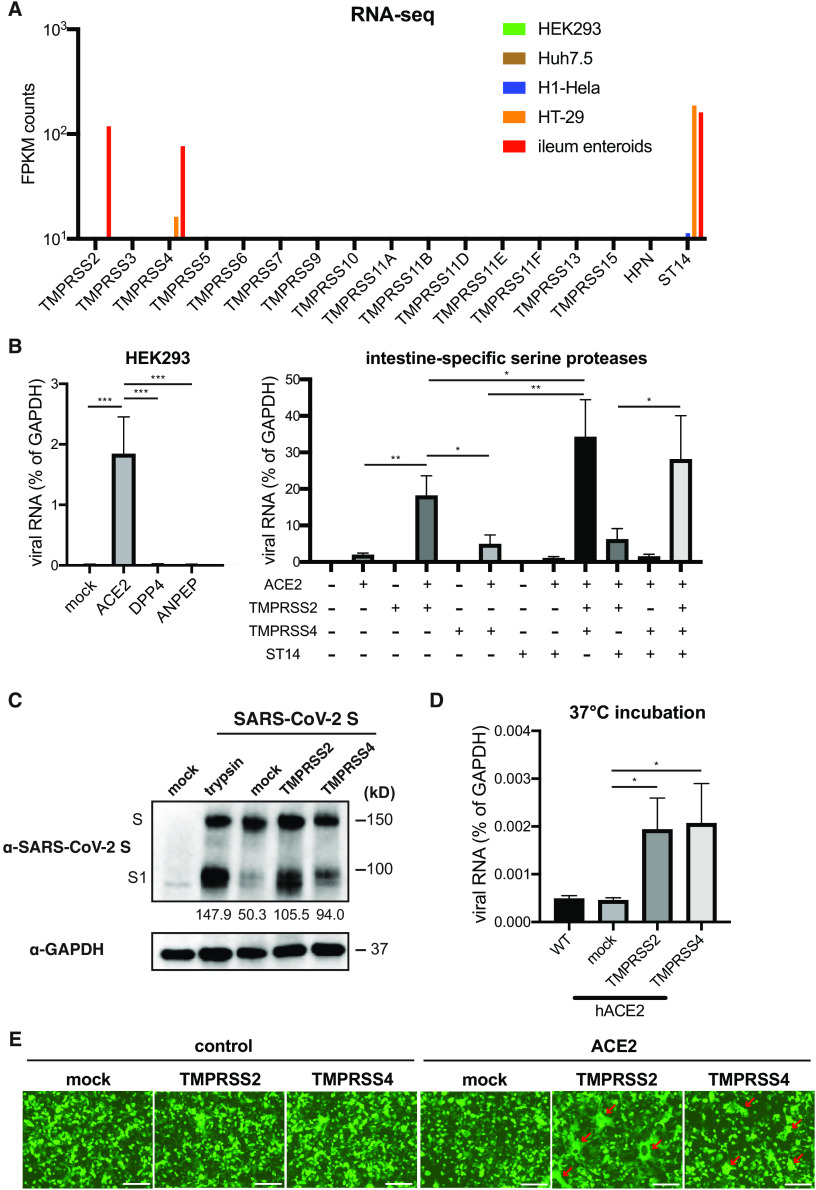
TMPRSS2, TMPRSS4 but not ST14 mediate SARS-CoV-2 S mediated entry. (A) Bulk RNA-sequencing results of intestine-specific serine protease expression in HEK293, Huh7.5, H1-Hela, HT-29 cells and human ileum enteroids. (B) HEK293 cells were transfected with pcDNA3.1-V5-ACE2, DDP4, or ANPEP for 24 hours (left panel), or transfected with indicated plasmid combination for 24 hours (right panel), and infected with 1.5X10^5^ PFU of VSV-SARS-CoV-2 for 24 hours. The expression of VSV-N was measured by RT-qPCR and normalized to that of *GAPDH*. (C) HEK293 cells stably expressing human ACE2 were transfected with SARS-CoV-2 S and TMPRSS2 or TMPRSS4 or 48 hours. Cells were treated with trypsin at 0.5 μg/ml for 10 min. The levels of S and GAPDH were measured by Western blot. The intensity of bands was quantified by ImageJ and shown as percentage of the bottom band versus the top band in each lane. (D) HEK293 cells stably expressing human ACE2 were transfected with TMPRSS2 or TMPRSS4 for 24 hours, incubated with 5.8X10^5^ PFU of VSV-SARS-CoV-2 on ice for 1 hour, washed with cold PBS for 3 times, and shifted to 37°C for another hour. The expression of VSV-N was measured by RT-qPCR and normalized to that of *GAPDH*. (E) Wild-type or human ACE2 expressing HEK293 cells were transfected with SARS-CoV-2 S and GFP, with or without TMPRSS2 or TMPRSS4 or 24 hours. The red arrows highlight the formation of large syncytia. Scale bar: 100 μm. For all figures except A, experiments were repeated at least three times with similar results. RNA-seq in Fig. 3A was performed once with duplicate samples. Data are represented as mean ± SEM. Statistical significance is indicated (*p≤0.05; **p≤0.01; ***p≤0.001).

Recent studies suggest that the membrane-bound TMPRSS2 protease cleaves the S protein and promotes SARS-CoV-2 entry (*3*, *6*, *33*). Two other serine proteases in the same family, TMPRSS4 and matriptase (encoded by *ST14*) were highly expressed in human IECs (Fig. 3A). Previous studies indicated that both TMPRSS4 and ST14 facilitate influenza A virus infection but neither had a role in SARS-CoV infection (*34*–*37*). In our mouse scRNA-seq dataset, TMPRSS4 and ST14 were expressed in ACE2^+^ mature enterocytes while TMPRSS2 was primarily expressed in ACE2^-^ secretory IECs (Fig. S3A).

To dissect the entry pathway of SARS-CoV-2 in IECs, we set up an ectopic expression system in HEK293 cells to evaluate VSV-SARS-CoV-2 infection. As reported (*2*, *5*), ACE2 conferred permissiveness to VSV-SARS-CoV-2 infection (Fig. 3B and S3B). Although TMPRSS2 alone did not mediate viral infection, co-expression of TMPRSS2 with ACE2 resulted in enhanced infectivity (Fig. 3B). Moreover, expression of TMPRSS4 but not ST14 also resulted in a significant increase in the levels of viral RNA and infectious virus in the presence of ACE2 (Fig. 3B and S3C). Co-expression of TMPRSS4 and TMPRSS2 had an additive effect and enabled maximal infectivity in cell culture (Fig. 3B and S3C-E).

We hypothesized that TMPRSS4, like TMPRSS2, might function as a cell surface serine protease to enhance S cleavage and promote viral entry. To test this idea, we co-expressed full-length SARS-CoV-2 S protein in an HEK293 cell line that stably expresses ACE2 with or without additional introduction of TMPRSS2 or TMPRSS4. In mock-transfected cells, we readily observed the full-length S and a cleaved product that corresponded to the size of S1 fragment (Fig. 3C), presumably cleaved by furin protease that is ubiquitously expressed (*38*). Compared to the positive control exogenous trypsin treatment, expression of TMPRSS2 or TMPRSS4 promoted S cleavage, as evidenced by the reduction of full-length S and increase levels of S1 protein (Fig. 3C).

Based on these results, we hypothesized that TMPRSS serine proteases facilitate virus infection by inducing S cleavage and exposing the fusion peptide for efficient viral entry. Indeed, TMPRSS4 expression did not affect virus binding at 4°C, but led to an increase in infection as evidenced by enhanced viral gene transcription 1 hour post warming cells to 37°C (Fig. 3D). We next determined the influence of TMPRSS2 or TMPRSS4 on SARS-CoV-2 S protein mediated cell-cell fusion. Previous work with SARS-CoV and MERS-CoV suggests that S mediated membrane fusion occurs in a cell-type dependent manner (*39*). Ectopic expression of S protein alone was sufficient to induce syncytia formation, independent of virus infection (Fig. 3E). This process was dependent on expression of both ACE2 and TMPRSS serine proteases. TMPRSS4 expression triggered S-mediated cell-cell fusion, although to a lesser extent than TMPRSS2 (Fig. 3E). These studies show that TMPRSS2 and TMPRSS4 activate SARS-CoV-2 S and enhance membrane fusion.

### TMPRSS2 and TMPRSS4 promote SARS-CoV-2 infection in enteroids

**Fig. 4:**
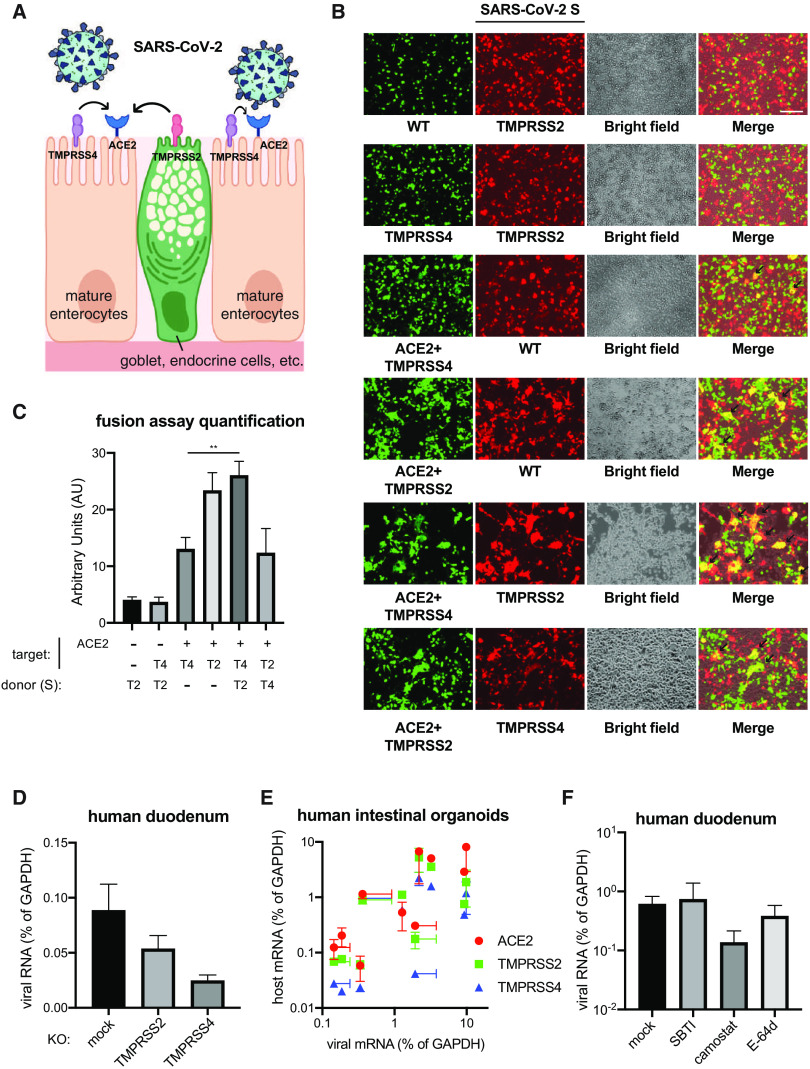
TMPRSS2 and TMPRSS4 promote VSV-SARS-CoV-2 infection in enteroids. (A) Schematic diagram of SARS-CoV-2 infection of human mature enterocytes. SARS-CoV-2 particles, host proteins, and host cells are not proportional to the true sizes. Based on the scRNA-seq data (Fig. S3A), TMPRSS4 is more highly expressed than TMPRSS2 on mature absorptive enterocytes and TMPRSS2 is more highly expressed on secretory cells than TMPRSS4. (B) GFP-expressing HEK293 cells were mixed at 1:1 ratio and co-cultured with HEK293 cells expressing SARS-CoV-2 S and TdTomato for 24 hours (right panel). Note the formation of cell-cell fusion (yellow), highlighted by black arrows. (C) Images in (B) were quantified based on the intensity of yellow signals. T2: TMPRSS2, T4: TMPRSS4. (D) Human duodenum enteroids in 3D Matrigel were transduced with lentiviruses encoding Cas9 and sgRNA against TMPRSS2 or TMPRSS4 (oligonucleotide information in Table S1). Gene knockout enteroids were seeded into monolayers and infected with 1.5X10^5^ PFU of VSV-SARS-CoV-2 for 24 hours. The expression of VSV-N was measured by RT-qPCR and normalized to that of GAPDH. (E) Human duodenum, ileum, and colon enteroids were infected with 2.9X10^5^ PFU of VSV-SARS-CoV-2 for 24 hours. The levels of indicated viral and host transcripts were measured by RT-qPCR and normalized to that of *GAPDH*. (F) Human duodenum enteroids seeded into collagen-coated 96-well plates were differentiated for 3 days, pre-treated with 50 μg/ml of soybean trypsin inhibitor (SBTI), 10 μM of camostat mesylate, or 10 μM of E-64d for 30 min, and infected with 1.5X10^5^ PFU of VSV-SARS-CoV-2 for 24 hours. The expression of VSV-N was measured by RT-qPCR and normalized to that of *GAPDH*. All experiments were repeated at least three times with similar results. Data are represented as mean ± SEM. Statistical significance is indicated (*p≤0.05; **p≤0.01; ***p≤0.001).

To further probe the mechanism of action of TMPRSS serine proteases, we designed an in vitro co-culture system where ACE2 and S were expressed on target and donor cells respectively. Based on the scRNA-seq data (Fig. S3A), we constructed an HEK293 cell line that co-expressed ACE2 and TMPRSS4 to mimic mature enterocytes and another HEK293 cell line that expressed TMPRSS2 to mimic goblet or other secretory IEC types (Fig. 4A, *left panel*). Target cells were transfected with GFP and mixed at 1:1 ratio with SARS-CoV-2 S containing donor cells that were transfected with TdTomato. TMPRSS4 could function *in cis*, i.e., on the same cells as ACE2, and induced fusion of GFP positive cells but not *in trans* (Fig. 4A, *right panel*). In comparison, TMPRSS2 could act both *in cis* and *in trans* and its expression on adjacent cells resulted in larger syncytia formation (Fig. 4A, *right panel*).

To determine the function of TMPRSS serine proteases in facilitating SARS-CoV-2 S mediated infection of primary human IECs, we used CRISPR/Cas9 gene editing to deplete TMPRSS2 or TMPRSS4 expression in human duodenum enteroids. Efficient knockout was confirmed by immunoblotting (Fig. S4A). Abrogation of TMPRSS4 expression led to a 4-fold reduction in VSV-SARS-CoV-2 replication in human enteroids, greater than seen for TMPRSS2 knockout cells (Fig. 4B). We examined the ACE2, TMPRSS2, and TMPRSS4 mRNA levels in a panel of human duodenum, ileum, and colon intestinal enteroids from different donors and correlated the susceptibility to VSV-SARS-CoV-2 infection (Fig. 4C). Higher ACE2 levels correlated with infection although TMPRSS2 and TMPRSS4 expression also showed a positive correlation as well (Fig. S4B). ACE2, TMPRSS2, and TMPRSS4 mRNA levels were not altered by VSV-SARS-CoV-2 infection (Fig. S4C).

In parallel, we also tested the effect of pharmacological inhibition of TMPRSS serine proteases on virus replication. We pretreated enteroids with camostat mesylate, a selective inhibitor of TMPRSS over other serine proteases including trypsin, prostasin and matriptase (*40*). Whereas camostat treatment significantly inhibited VSV-SARS-CoV-2 infection, soybean trypsin inhibitor (SBTI) and E-64d, a cysteine protease inhibitor that blocks cathepsin activity and inhibits SARS-CoV-2 infection in Vero cells (*3*), did not affect virus replication in enteroids (Fig. 4D). Taken together, our data support a model in which SARS-CoV-2, at least in TMPRSS^+^ human IECs, is more reliant on the membrane-bound serine proteases (Fig. 4A, *left panel*) than SARS-CoV and other CoVs and can efficiently utilize both TMPRSS2 and endosome-localized cathepsins as alternative routes of entry (*41*, *42*).

### SARS-CoV-2 is rapidly inactivated in the human GI tract

**Fig. 5:**
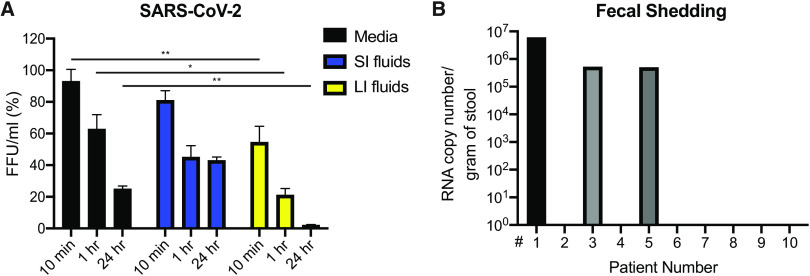
SARS-CoV-2 rapidly lose infectivity in the human GI tract. (A) 2.5X10^5^ PFU of SARS-CoV-2-mNeonGreen virus was incubated with M199 media, simulated human small intestinal (SI) fluid, or simulated human large intestinal (LI) fluid for indicated time points at 37°C. The virus was subsequently serially diluted and added to MA104 cells for 24 hours and GFP signals were scanned by Typhoon 5. (B) Stool specimens from 10 COVID-19 patients were collected and subjected to QPCR experiments to quantify the absolute levels of SARS-CoV-2 N gene. Figure 5A was performed once in quadruplicate. Figure 5B was performed once. Data are represented as mean ± SEM. Statistical significance is indicated (*p≤0.05; **p≤0.01; ***p≤0.001).

Our results suggest that SARS-CoV-2 viruses can infect human IECs apically and are released via the apical route into the lumen (Fig. 2). Human enteric viruses that spread via the fecal-oral route typically withstand the harsh environment in the GI tract, including the low pH of gastric fluids, bile and digestive enzymes in the small intestine, dehydration and exposure to multiple bacterial byproducts in the colon. We investigated the stability of a recombinant SARS-CoV-2 mNeonGreen reporter virus (*43*) in different simulated human gastric and intestinal fluids. Compared to rotavirus, which is transmitted by the fecal-oral route (*44*), SARS-CoV-2 lost infectivity in the low pH simulated gastric fluid at 10 min post incubation (Fig. S5A). However, there was residual SARS-CoV-2 virus in simulated human small intestinal fluid that contains biological surfactants including taurocholic acid sodium salt and lecithin (Fig. 5A). In contrast, SARS-CoV-2 was inactivated by certain components in the simulated human colonic fluids (Fig. 5A). The virus titers decreased by 5-fold within 1 hour, and little infectious virus was detectable at the 24-hour time point (Fig. 5A). In contrast, rotavirus remained stable in all simulated gastric and enteric fluids tested (Fig. S5A-B).

Combined with the human enteroid results (Fig. 1-2), we reasoned that the SARS-CoV-2 replicates in human IECs but may then be rapidly inactivated in the lumen of the colon. We collected stool specimens from a small group of COVID-19 patients. From 3 out of 10 fecal samples, we detected high RNA copy numbers of SARS-CoV-2 viral genome (Fig. 5B). However, we were unable to recover any infectious virus using a highly sensitive cell-based assay (*6*) as compared to the media spiked with cell culture-derived wild-type SARS-CoV-2 viruses (Fig. S5C).

## Discussion

In this study we addressed an important basic and clinically relevant question: Is the persistent viral RNA seen in COVID-19 patients’ stool infectious and transmissible? We showed that despite SARS-CoV-2’s ability to establish robust infection and replication in human IECs (Fig. 1-2), the virus is rapidly inactivated by simulated human colonic fluid and a limited number of viral RNA-positive fecal samples did not contain infectious virus. Thus, the large quantities of viral RNA that transit through the GI tract and shed into the feces may not carry substantial infectious risk. Due to the limitation of our sample size, we cannot definitely conclude that fecal-oral transmission of COVID-19 does not occur. Another potential caveat is that the sample collection depends on the transit time. In other words, if COVID-19 patients have rapid transit colonic motility due to either small bowel or colon pathophysiology or in the setting of diarrhea, the feces may still contain residual infectivity. However, our data are consistent with the previous reports from SARS-CoV and MERS-CoV publications which concluded that despite long duration of viral RNA shedding, no infectious virus could be recovered from the patients’ feces (*45*, *46*).

Intriguingly, the simulated small intestinal fluid, which contains taurocholic acid sodium salt and lecithin, important surfactant components of the bile, did not inactivate virus as expected (Fig. 5A). Further experiments are being performed with bile acids and bile salts that act as detergents in gut. Recent studies showed a heavy glycosylation of SARS-CoV-2 S protein (*47*) and it is possible the glycan coating confers some stability against the enzymatic digestion and bile salt solubilization. Two recent publications demonstrated that SARS-CoV-2 has unique stability in the laboratory environment (*48*, *49*). Further work is needed to determine which bioactive component(s) of the simulated colonic fluid that are absent from small intestinal fluid may function to inactivate virus in vivo.

Recent work from multiple groups has shown a key role of TMPRSS2 in cleaving SARS-CoV-2 S protein. We provide data that besides TMPRSS2, TMPRSS4 also increases SARS-CoV-2 infectivity, at least in gut epithelial cells (Fig. 3-4). We observed an intriguing additive effect of the two enzymes, as both target single arginine or lysine residues. We speculate that this synergy stems from a distinct cellular and subcellular localization in neighboring cells (Fig. 4A). Future work with recombinant proteins and enzymatic digestion may help address whether S cleavage is sequential and whether the two enzymes act on different sites on the S protein.

Finally, we observed syncytia formation in human enteroids. These results may have implications for virus cell-cell spread and evasion of antibody neutralization (*50*). During the revision of this work, two manuscripts were published that examined SARS-CoV-2 infection of human IECs (*51*, *52*). Cell fusion also was observed, although it was not discussed. It is unclear at this point whether the fused IECs will still undergo anoikis or an alternative accelerated cell death, which may cause a breach in barrier integrity and give rise to some of the GI pathology and symptomatology seen in COVID-19 patients. It is possible that in the small intestine additional proteases such as trypsin enhance viral infection and pathogenesis by triggering more robust IEC fusion. Although viruses are not released into the basolateral compartment, a leaky gut might allow virus to disseminate to other systemic organs including the lung and liver. This hypothesis, while speculative, is consistent with the clinical observation that in some COVID-19 patients, the GI symptoms precede the respiratory illness (*8*, *9*). Whether the gut serves as a primary site of infection and contributes to systemic diseases in individual patients requires further study.

## Materials and Methods

### Study Design

The goal of this study was to examine the possibility of SARS-CoV-2 replication in the human intestine and potential fecal-oral transmission. A number of approaches and techniques, including single-cell RNA-sequencing, human intestinal organoids, VSV-SARS-CoV-2-S-GFP chimeric virus, and wild-type SARS-CoV-2 virus were employed. The numbers of experimental and technical replicates in each group and the statistical analysis applied are described in the figure legends and Statistical Analysis section.

### Plasmids, Cells, Reagents, and Viruses

Plasmids: Human ACE2, DPP4, ANPEP, TMPRSS2, TMPRSS4, and ST14 were cloned into a pcDNA3.1/nV5-DEST vector with an N-terminal V5 tag and a neomycin selection marker. Human TMPRSS2 and TMPRSS4 were also cloned into a pLX304 lentiviral vector with a C-terminal V5 tag and a blasticidin selection marker. pEGFP-N1 and pCMV-TdTomato were commercially purchased from Clontech. Codon-optimized SARS-CoV-2 S was a kind gift from Dr. Nevan J. Krogan at UCSF (*53*).

Cells: African Green Monkey kidney epithelial cell lines MA104 (CRL-2378.1) and human embryonic kidney cell line HEK293 (CRL-1573) were obtained from American Type Culture Collection (ATCC) and cultured in complete M199 medium and complete DMEM medium, respectively. HEK293 cells were transfected with V5-ACE2 plasmid, V5-TMPRSS2 plasmid, or transduced with TMPRSS2-expressing lentivirus. HEK293 cells stably expressing either human ACE2 or TMPRSS2 were selected under 500 μg/ml G418. HEK293 cells stably expressing both human ACE2 and TMPRSS2 were selected under 500 μg/ml G418 and 5 μg/ml blasticidin.

Reagents: Simulated human gastric fluid (Fasted State Simulated Gastric Fluid/FaSSGF, pH 1.6), simulated human small intestinal fluid (Fasted State Simulated Intestinal Fluid/FaSSIF-V2, pH 6.5), and simulated human colonic fluid (Fasted State Simulated Colonic Fluid/FaSSCoF, pH 7.8) were purchased from BioRelevant, UK and reconstituted based on the manufacturer’s instructions. Soybean trypsin inhibitor (SBTI), camostat mesylate, and E-64d were purchased from Selleckchem. Trypsin from porcine pancreas was purchased from Sigma-Aldrich. Primary antibodies used in this study included: CD26 (MA2607, Thermo Fisher); GAPDH (631402, Biolegend); GFP (2555S, Cell Signaling); SARS-CoV-2-S (S1) (PA5-81795, Thermo Fisher); TMPRSS2 (sc-515727, Santa Cruz); TMPRSS4 (sc-376415, Santa Cruz); and V5 (13202S, Cell Signaling).

Viruses: VSV-SARS-CoV-2 GFP reporter chimeric virus was constructed by P.W. Rothlauf and S.P.J. Whelan using the SARS-CoV-2 Wuhan-Hu-1 spike and the VSV Indiana strain. The full sequence and a detailed characterization of this chimeric virus will be published separately. The virus was propagated in MA104 cells in T175 flasks. SARS-CoV-2 strain 2019-nCoV/USA-WA1/2020 was obtained from Natalie Thornburg at the Centers for Disease Control and Prevention. A mNeonGreen SARS-CoV-2 reporter virus was used as previously reported (*43*). Virus was passaged once in Vero CCL81 cells (ATCC) and titrated by focus-forming assay in Vero-E6 cells. Plaque assays were performed in MA104 cells seeded in 6-well plates using an adapted version of the rotavirus plaque assay protocol (*23*). Human rotavirus WI61 strain was propagated as described before (*54*). TCID50 and FFU assays were performed in MA104 cells seeded in flat-bottom 96-well plates using serial dilutions. Virus infections were performed with the initial inoculum removed at 1 hour post adsorption.

### Human intestinal enteroids

One duodenum (#CD94), five ileum (#14-75, #211D, #262D-2, #265D, #251D-2), and one colon (#235A) enteroids were used in this study. All enteroids were derived from de-identified tissue from healthy subjects without inflammatory bowel disease who were undergoing colonoscopy and provided informed consent at Stanford University or Washington University School of Medicine. In brief, enteroids were cultured in maintenance media (advanced DMEM/F12 media supplemented with L-WRN-conditioned media that contains Wnt-3a, R-spondin 3, and Noggin) in 3D Matrigel in 24-well plates (*55*). For passing enteroids, ROCK inhibitor Y-27632 and GSK-3 inhibitor CHIR99021 were added. For differentiation, conditioned media was replaced with 50% Noggin in the form of recombinant proteins. Enteroids, when required, were digested into single cells using TrypLE (Thermo Fisher) and seeded into collagen-coated Transwells (0.33 cm^2^, 0.4 μm pore size, Polycarbonate, Fisher #07-200-147) in Y-27632-supplemented maintenance media in 24-well plates. TEER measurement was performed using the Millicell ERS-2 Voltohmmeter (Millipore). Enteroids in monolayers were used for virus infection if the cultures had TEER greater than 1000 Ω.cm2 at 5 days post seeding. For CRISPR/Cas9 knockout, enteroids in 3D Matrigel were digested with 5 mM EDTA and transduced with lentiviral vectors encoding Cas9 and single-guide RNA against TMPRSS2 or TMPRSS4 (see Table S1) in the presence of polybrene (8 μg/ml). At 48 hours post transduction, puromycin (2 μg/ml) was added to the maintenance media. Puromycin was adjusted to 1 μg/ml upon the death of untransduced control enteroids.

### RNA extraction and quantitative PCR

Total RNA was extracted from cells using RNeasy Mini kit (Qiagen) and reverse transcription was performed with High Capacity RT kit and random hexamers as previously described (*56*). QPCR was performed using the AriaMX (Agilent) with a 25 μl reaction, composed of 50 ng of cDNA, 12.5 μl of Power SYBR Green or Taqman master mix (Applied Biosystems), and 200 nM both forward and reverse primers. All SYBR Green primers and Taqman probes used in this study are listed in Table S1.

### Bright-field and immunofluorescence microscopy

For brightfield and epifluorescence, cultured cells or human enteroids were cultured in 24-well plates and images were taken by REVOLVE4 microscope (ECHO) with a 10X objective. Co-localization signals were quantified by ImageJ plugins and geotests.net. For confocal microscopy, enteroids were seeded into 8-well Nunc chamber slides (Thermo Fisher), cultured under maintenance or differentiation conditions, infected with VSV-SARS-CoV-2, and fixed with 4% paraformaldehyde as previously described (*54*). Samples were then stained with the following primary antibodies or fluorescent dyes: ACE2 (sc-390851 AF594, Santa Cruz), DAPI (P36962, Thermo Fisher), SARS-CoV-2 S (CR3022 human monoclonal antibody (*57*)), villin (sc-58897 AF488, Santa Cruz), and phalloidin (Alexa 647-conjugated, Thermo Fisher). Stained cells were washed with PBS, whole-mounted with Antifade Mountant, and imaged with Zeiss LSM880 Confocal Microscope at the Molecular Microbiology imaging core facility at Washington University. Z-stack was applied for imaging 3D human enteroids. Images were analyzed by Volocity v6.3 (PerkinElmer) and quantification was determined by CellProfiler (Broad Institute).

### Single-cell RNA-sequencing analysis

Five-day -old 129/Sv strain suckling mice were obtained from the Greenberg lab breeding colony at Stanford University. IECs were isolated from small intestine tissue pooled from 2 males and 1 female by EDTA-DTT extraction method and cell sorting was performed as previously described (*23*, *58*). Cells were stained with LIVE/DEAD Aqua Dead Cell Stain Kit (Thermo Fisher) and live cells were sorted using a BD FACSAria II flow cytometer. Preparation of single cell suspensions was performed at the Stanford Genome Sequencing Service Center (GSSC). Cell suspensions were processed for single-cell RNA-sequencing using Chromium Single Cell 3′ Library and Gel Bead Kit v2 (10X Genomics, PN-120237) according to 10X Genomics guidelines. Libraries were sequenced on an Illumina NextSeq 500 using 150 cycles high output V2 kit (Read 1-26, Read2-98 and Index 1-8 bases). The Cell Ranger package (v3.0.2) was used to align high quality reads to the mouse genome (mm10). Normalized log expression values were calculated using the scran package (*59*). Highly variable genes were identified using the FindVariableGenes function (Seurat, v2.1) as described (*60*). Batch effects from technical replicates were removed using the MNN algorithm as implemented in the batchelor package’s (v1.0.1) fastMNN function. Imputed expression values were calculated using a customized implementation (https://github.com/kbrulois/magicBatch) of the MAGIC (Markov Affinity-based Graph Imputation of Cells) algorithm (*61*) and optimized parameters (t = 2, k = 9, ka = 3). Cells were classified as dividing or resting using a pooled expression value for cell cycle genes (Satija Lab Website: regev_lab_cell_cycle_genes). For UMAP and tSpace embeddings, cell cycle effects were removed by splitting the data into dividing and resting cells and using the fastMNN function to align the dividing cells with their resting counterparts. Dimensionality reduction was performed using the UMAP algorithm and nearest neighbor alignments for trajectory inference and vascular modeling were calculated using the tSpace algorithm. Pooled expression of all viral genes was calculated using the AddModuleScore function from the Seurat package.

### Human IRB Approval

COVID-19 patient derived biospecimens (stool) were collected with an IRB approved protocol with written informed consent (Institutional Review Board #202003085) by the Washington University Human Research Protection Office and under the management of the Institute of Clinical and Translational Sciences. The collection and use of human tissue for establishing primary epithelial cell culture or organoid culture was approved by the Washington University Human Research Protection Office (Institutional Review Board #201404112) and collected with written informed consent by the Washington University Digestive Diseases Research Core Center Biobank Core.

### Statistical Analysis

All bar graphs were displayed as means ± SEM. Statistical significance in data in Fig. 2C was calculated by Student's *t* test using Prism 8.4.1 (GraphPad). Statistical significance in data Fig. 1C, 1E, 2A, 2D, 3B, 3D, 4A, 5A, S2B, and S3C was calculated by pairwise ANOVA using Prism 8. Simple linear regression was performed to calculate R squared and p values for Fig. S4B. Statistical significance of all data are presented as asterisks (*p≤0.05; **p≤0.01; ***p≤0.001). All experiments other than Fig. 1A, 2C, 2D, 2E, 5A, 5B, S5A, and S5C have been repeated at least three times. The single-cell RNA-seq analysis (Fig. 1A and S3A) was performed once using small intestinal tissue pooled from three mice. Bulk RNA-seq analysis (Fig. 3A and S1C) was performed once for each cell type using two duplicate samples.

## Supplementary Materials

https://immunology.sciencemag.org/cgi/content/full/5/47/eabc3582/DC1 Fig. S1. ACE2 is highly expressed in the human intestine Fig. S2. VSV-SARS-CoV-2 induces syncytia formation in cell culture and human intestinal cells Fig. S3. TMPRSS2 and TMPRSS4 enhance VSV-SARS-CoV-2 infectivity Fig. S4. Validation of CRISPR/Cas9 mediated knockout of TMPRSS2 and TMPRSS4 in human enteroids Fig. S5. Rotavirus remains infectious in simulated human gastric and intestinal fluids Table S1. QPCR and CRISPR deletion oligonucleotide information. Table S2. Bulk RNA-sequencing results of HEK293, Huh7.5, H1-Hela, HT-29 cells, and human ileum enteroids (Excel spreadsheet). Table S3. Raw data file (Excel spreadsheet). Video S1. Formation of syncytia in 3D human duodenum enteroids infected by SARS-CoV-2 GFP chimeric virus.
